# Effect of Vitamin D Supplementation on Risk of Breast Cancer: A Systematic Review and Meta-Analysis of Randomized Controlled Trials

**DOI:** 10.3389/fnut.2021.655727

**Published:** 2021-04-01

**Authors:** Ziyi Li, Liangzhi Wu, Junguo Zhang, Xin Huang, Lehana Thabane, Guowei Li

**Affiliations:** ^1^Centre for Clinical Epidemiology and Methodology, Guangdong Second Provincial General Hospital, Guangzhou, China; ^2^Department of Gynecology, Guangdong Second Provincial General Hospital, Guangzhou, China; ^3^Department of Health Research Methods, Evidence, and Impact, McMaster University, Hamilton, ON, Canada; ^4^St. Joseph's Healthcare Hamilton, Hamilton, ON, Canada

**Keywords:** breast cancer, mammographic density, systematic review, vitamin D, meta-analysis

## Abstract

**Objective:** Laboratory findings indicated that vitamin D might have a potent protective effect on breast cancer, but epidemiology studies reported conflicting results. The aim of the study was to conduct a systematic review and meta-analysis to clarify the efficacy of vitamin D supplementation on risk of breast cancer.

**Methods:** MEDLINE, EMBASE, The Cochrane Central Register of Controlled Trials, ClinicalTrials.gov, and abstracts of three major conferences were searched (up to December 8, 2020). Parallel randomized controlled trials (RCTs) examining the efficacy of vitamin D supplementation on risk of breast cancer or change of mammography compared with placebo in females were included. Data were meta-analyzed using a random-effects model. Bayesian meta-analysis was conducted to synthesize the results using data from observational studies as priors.

**Results:** Seven RCTs were identified for effect of vitamin D on risk of breast cancer, with 19,137 females included for meta-analysis. No statistically significant effect of vitamin D on risk of breast cancer was found in classical random-effects meta-analysis (risk ratio = 1.04, 95% confidence interval: 0.84–1.28, *p* = 0.71). When Bayesian meta-analyses were conducted, results remained non-significant. There was no statistically significant effect of vitamin D on mammography density observed: mean difference = 0.46, 95% confidence interval: −2.06 to 2.98, *p* = 0.72.

**Conclusion:** There is insufficient evidence to support the efficacy of vitamin D supplementation in breast cancer risk and change of mammography density. The protective effect of vitamin D on risk of breast cancer from previous observational studies may be overestimated.

**Systematic Review Registration:** PROSPERO, identifier CRD42019138718.

## Introduction

Breast cancer is one of the most commonly occurring cancers and is among the leading causes of death in women worldwide (1). Although advances in early detection and treatment have decreased the mortality, incidence rates of breast cancer are still increasing in most countries during the past two decades (2). Breast cancer afflicts 2.1 million women each year currently; and it is estimated that by 2050, there will be 3.2 million new cases each year approximately (3, 4). Developing effective primary prevention strategies to reduce the incidence rates therefore remains to be of high priority.

Vitamin D is the precursor to the hormone calcitriol (1,25-dihydroxyvitamin D3), which regulates numerous actions in many tissues of the human body (5). Vitamin D can be produced endogenously in the skin by sun exposure and be obtained from diet and supplements to a minor extent as well. Vitamin D insufficiency, defined as serum 25-hydroxyvitamin D (25[OH]D) lower than 25 ng/ml, has been reported in many regions with high prevalence, especially in high-latitude countries. It is anticipated that one billion people have vitamin D deficiency globally (6). Vitamin D is well-known for its role in maintaining calcium homeostasis and mediating bone mineralization (7, 8). Besides its benefits to bone health, the effects in the prevention and treatment of a variety of diseases such as cancer, autoimmune disorders, and cardiovascular disease had been investigated in recent research (9–11). Laboratory findings indicate that vitamin D may have potent anticancer effects such as anti-proliferative, pro-apoptotic, pro-differentiating, anti-inflammatory, anti-invasion, and anti-angiogenesis effects (5, 9, 12–17). The links between higher vitamin D levels, including vitamin D intake and serum 25(OH)D, and reduced risk of breast cancer had been revealed by observational epidemiology studies (18–21). A recent meta-analysis that included 22 observational studies reported that higher vitamin D intake was significantly associated with decreased risk of breast cancer (22). Randomized controlled trials (RCTs) exploring the effect of vitamin D on breast cancer has been published; however, results from these trials were not consistent with observational studies. In 2014, a meta-analysis combining seven RCTs concluded that vitamin D supplementation may have a protective effect on breast cancer but without statistical significance [risk ratio (RR) = 0.97, 95% confidence interval (CI): 0.86 to 1.09] (23).

Mammographic breast density [mammography density (MD)] is one of the strongest indicators of breast cancer (24–29). It is defined as the proportion of fibroglandular tissue within the whole breast (30), reflecting the extent of epithelial and non-epithelial cells in the breast as well as epithelial and/or stromal proliferation (31, 32). MD is being increasingly considered in guiding personalized screening recommendations (33, 34). In experimental studies, vitamin D was reported to potentially decrease MD by inhibiting both the synthesis and the biological actions of estrogens (35–37). However, both observational studies and RCTs found conflicting results on this topic (38–45).

Given the inconsistent findings and more recent research published, we conducted a systematic review and meta-analysis to summarize the most up-to-date evidence from RCTs, aiming to clarify the effect of vitamin D supplementation on risk of breast cancer. Our outcomes included risk of breast cancer and MD.

## Materials and Methods

The systematic review was conducted according to the Cochrane Handbook for Systematic Reviews of Interventions (46). We summarized the identification, screening, and inclusion of studies according to the Preferred Reporting Items for Systematic Reviews and Meta-Analyses (PRISMA) diagram (47). The review was registered on PROSPERO (Identifier: CRD42019138718).

### Search Strategy

MEDLINE, EMBASE, The Cochrane Central Register of Controlled Trials (CENTRAL), and ClinicalTrials.gov were searched by two reviewers (ZL and LW, up to September 2019). Moreover, an updated search was performed up to December 8, 2020. We used descriptors that include synonyms of vitamin D, breast cancer, MD, and RCT in different combinations (descriptors can be found in Supplementary Table 1). No limitations of language or publication status were added to our searches. The reference list of articles and other reviews retrieved in the search were also searched for relevant articles.

Unpublished studies were identified by searching the abstract of top three conferences in the area of breast cancer: St. Gallen International Breast Cancer Conference (https://www.oncoconferences.ch/Breast-Cancer-Conferences), American Society of Clinical Oncology Annual Meeting (https://meetings.asco.org/am/attend), and San Antonio Breast Cancer Symposium (https://www.sabcs.org/). Missing data were sought from the original authors or from secondary publications of the same study.

### Study Eligibility

Parallel RCTs investigating the effect of vitamin D supplementation on risk of breast cancer or MD in adult females aged 18 years or over at baseline were eligible for inclusion. Participants who had history of breast cancer and have abnormalities such as hyperparathyroidism were excluded. To meet our criteria, at least one of the intervention arms had to include intake of vitamin D supplementation as an intervention. Studies involving vitamin D as intervention at any dose, duration, and frequency were eligible. The cointerventions were allowed only if they were used equally in all arms of the study so that the effect of vitamin D could be isolated. Only trials using placebos in their control groups were included.

### Outcome

Our primary outcome was risk of breast cancer. The secondary outcome was the change in MD from baseline. The outcomes were measured as defined by the individual included studies.

### Data Collection

Two reviewers (ZL and LW) independently screened and selected studies for possible inclusion in the study. Any disagreement was resolved by consensus, and a third reviewer (GL) was consulted if disagreement persisted. Agreement between authors was quantified using the kappa statistic.

For studies that fulfilled our inclusion criteria, two reviewers (ZL and LW) independently extracted data using a especially developed data extract form, which was designed and piloted prior to its use. The following information was extracted: (1) participant characteristics: age, status of menopause, baseline levels of serum 25(OH)D, baseline MD (for secondary outcome), total sample size, number of female participants, and study settings including countries and latitude; (2) intervention: number of arms, sample size of each arm, randomization and allocation concealment method, blinding, dose and type of supplementation (D2 or D3), frequency and duration of intervention, cointervention in each arm, and withdrawals and drop-offs; and (3) outcome measures: description of measures used, incidence of breast cancer, and measures of MD. Any disagreement was resolved by discussion and consensus among all the review authors.

### Statistical Analysis

#### Main Analysis

According to our protocol, a random-effects model was performed to synthesize the data by pooling the results of the included studies. We analyzed the data using Review Manager (RevMan) V5.2 for Windows (the Nordic Cochrane Center, the Cochrane Collaboration, Copenhagen, Denmark). We calculated the pooled RR for dichotomous data (breast cancer risk) and the weighted mean difference (WMD) for continuous data (MD) measured on the same scale. Heterogeneity between included studies was assessed using the I^2^ statistic, with *I*^2^ > 50% or *p* < 0.1 taken as implying significant heterogeneity.

Additionally, to incorporate the evidence from observational studies, we synthesized the results from the RCTs using a Bayesian random-effects model in conjunction with observational studies. Three prior distributions were applied to the Bayesian random-effects model: a “non-informative” prior distribution, an “informative” prior distribution, and a “skeptical” prior distribution. The latter two distributions were identified based on the pooled information from observational studies included in a recent meta-analysis that reported a pooled RR of 0.97 (95% CI: 0.95–1.0, *p* = 0.026) (22). By comparing these three priors, we could have a sense of (1) whether treating the information from observational studies could yield different findings, and (2) if so, whether observational studies were likely to over- or underestimate the causal relationship after combining the evidence from RCTs (48). Posterior distributions were obtained by incorporating data from RCTs into prior distributions. Considering the computational burden and tractability, we used 100,000 Markov chain Monte Carlo cycles with two chains of simulations, a burn-in of 10,000, and a thin of 10 (49). Convergence, assessed using the Gelman–Rubin statistic, was approached if the Gelman–Rubin statistic tended to 1 (50). The autocorrelation was assessed based on the autocorrelation function plots. The intervention efficacy was estimated by the RR, which was estimated through 95% credible intervals (CrIs). We conducted a sensitivity analysis with different prior distributions for between-study variance or SD (i.e., gamma distribution for between-study variance and uniform distribution for between-study SD) to evaluate the robustness of the results of the Bayesian analyses. WinBUGS 1.4 (MRC Biostatistics Unit, Cambridge, UK) was used for Bayesian analysis.

#### Subgroup Analysis

For primary outcome, the heterogeneity was examined by carrying out the following subgroup analysis: (1) different vitamin D dosages (less than median dose vs. no less than median); (2) different durations of intervention (less than median duration vs. no less than median); (3) different menopausal status of the participants (post- vs. pre-); (4) different latitudes where the studies were carried out (lower than median dose vs. no less than median); and 5) different serum 25(OH)D levels of the participants at baseline (less than median vs. no less than median).

#### Sensitivity Analysis

We conducted three sensitivity analyses using random-effects models for risk of breast cancer by (1) excluding studies that were rated as at high risk of bias; (2) excluding studies that involved non-study cointerventions; and (3) using the data from the shortest study period in each study.

#### Quality Assessment

The risk of bias for each included studies was examined by Cochrane Collaboration “risk of bias” assessment tool (46), which included evaluations of random sequence generation, allocation concealment, blinding of participants and personnel, blinding for outcome assessment, incomplete outcome data, selective reporting, and other sources of bias. The quality of a body of evidence across all studies for each outcome was assessed using the Grading of Recommendations Assessment, Development and Evaluation (GRADE) tool (51) with GRADEprofiler software V.3.6.

#### Risk of Publication Bias

Potential risk of publication bias was examined by the construction of funnel plots, Begg's rank correlation test, and Egger's regression test.

## Results

### Study Identification

**Figure 1:**
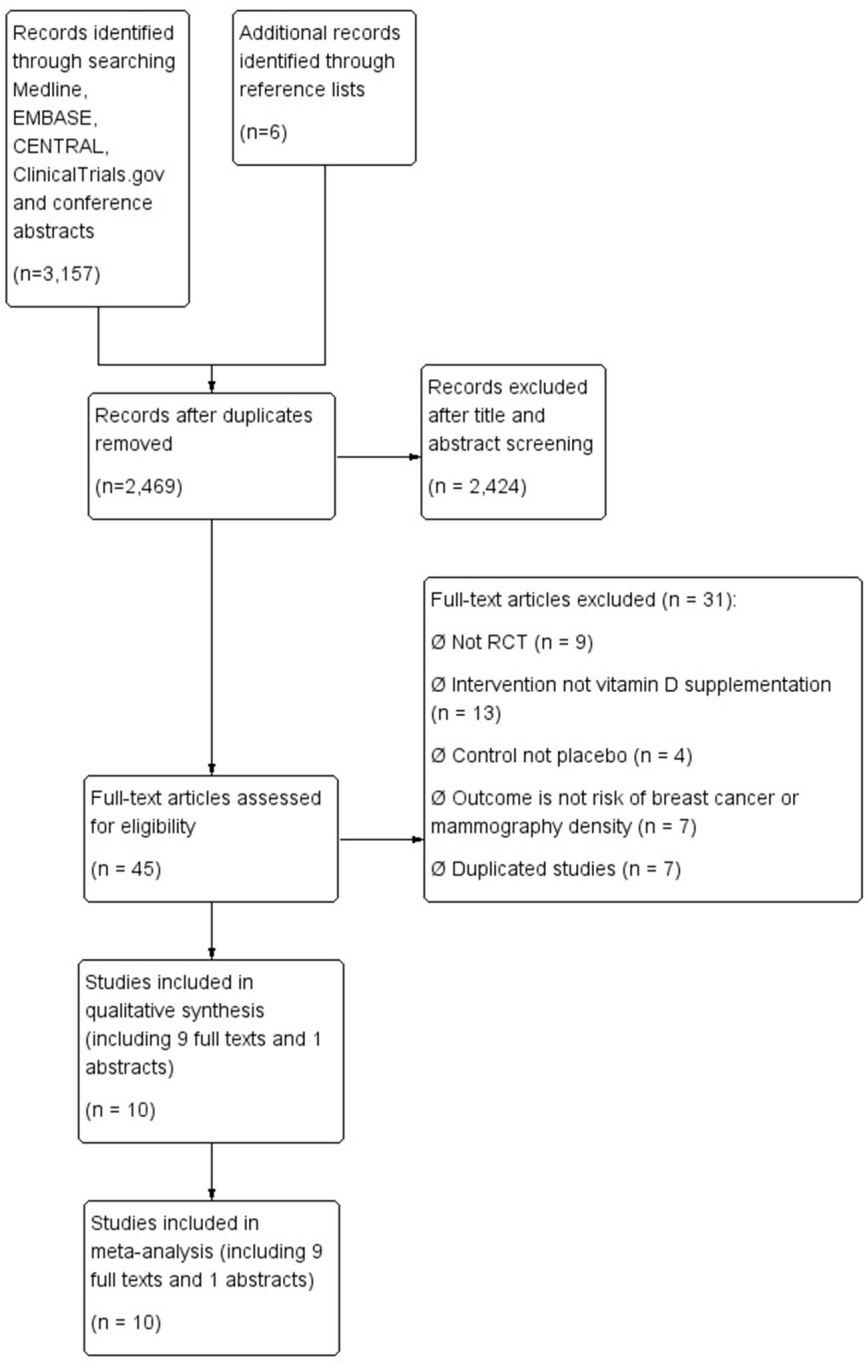
Flow diagram for selection process.

We identified 3,157 citations from the literature search and reference lists, from which 688 duplicates were removed, leaving 2,469 citations that remained for title and abstract screening. Forty-five citations were retrieved for full-text screening. There were eight discrepancies resolved by discussion between reviewers (unweighted kappa = 0.89; 95% CI: 0.81–0.96). No further studies were identified from unpublished literature. Ten studies [nine full texts (39, 40, 52–58) and one abstract (38)] that met the inclusion criteria were included in the final meta-analyses (Figure 1 shows the flow diagram of study selection process).

### Characteristics of Included Studies

Characteristics of included studies are shown in Table 1. Among the 10 included RCTs, seven reported risk of breast cancer as outcome (52–58), while the other three reported MD (38–40). All studies were conducted in North America or Europe, with four in high-latitude countries (52, 53, 55, 56). A total of 34,150 participants (including 20,355 females) were randomized. The mean age of the participants ranged from 43 to 77 years. Five studies included post-menopause participants (52, 53, 55–57), three included pre-menopause (38–40), and the other two did not specify menopausal status (54, 58). The participants' mean serum 25(OH)D level at baseline ranged from 18 to 36.2 ng/ml. All the 10 studies used oral vitamin D3 (cholecalciferol) supplementation as intervention, three among which involved cointervention (55, 57, 58). The dosage of vitamin D varied from 800 to 3,300 IU/day roughly. The duration of follow-up ranged from 4 months to 6 years.

**Table 1:** Characteristics of included studies.

**References**	**Participants**						**Intervention**				**Outcome**
	**No. of randomized participants (No. of female)**	**Age, years, mean (SD)**	**Latitude**	**Menopausal status**	**Baseline serum 25(OH)D level**	**Baseline MD**	**Type of vitamin D, dose**	**Control group**	**Administration**	**Follow-up duration**	**Outcome measures**
**Incidence**											
Avenell et al. (55)	5,292 (4,481)	77 (6)	50–59°N (across UK)	Post	Vit D group had 32% participants at high risk of Vit D deficiency; placebo group had 31.6%	N/A	D3, 800 IU/day	Placebo, p + ca	Oral	4 months	N/A
Lappe et al. (57)	1,180 (1,180)	66.7 (7.3)	41.4°N	Post	Vit D + Ca group: 71.8 (20.0) nmol/L; Ca group: 71.6 (20.5) nmol/L	N/A	D3, vitamin D 1,100 IU/day and Ca 1,400–1,500 mg/day	Ca 1,400–1,500 mg/day	Oral	4 years	N/A
Larsen et al. (53)	112 (77)	61 (10)	56°N	Post	Vit D group: 23(9) ng/ml; placebo group: 23 (12) ng/ml	N/A	D3, 3,000 IU/day	Placebo	Oral	20 weeks	N/A
Manson et al. (58)	25,871 (13,085)	67.1 (7.1)	N/A	N/A	30.8 (10.0) ng/ml (77 nmol/L)	N/A	D3, vitamin D 2,000 IU/day or vitamin D 2,000 IU/day and n-3 fatty acid 1g/day	Placebo or placebo and n-3 fatty acid 1 g/day	Oral	Median follow-up of 5.3 years (range, 3.8–6.1)	Through the national health service databases
Murdoch et al. (54)	322 (241)	47	43°31′48″S	N/A	Vit D group: 9 (9) ng/ml; placebo group: 28 (9) ng/ml	N/A	D3, 200,000 IU for 1 month then 100,000 IU/month	Placebo	Oral	18 months	The medical record was examined
Witham et al. (52)	159 (77)	76.8	55.86 N	Post	Vit D group: 18 (6) ng/ml; placebo group: 18 (6) ng/ml	N/A	D3, 100,000 U/3 months	Placebo	Oral	12 months	Cancer was confirmed on the basis of histologic or cytologic data.14
Wood et al. (56)	305 (305)	Vit D group: 63.5 (1.9); 62.1 (2.3); placebo group: 63.9 (2.3)	57.15°N	Post	Vit D group: 32.74 (12.9), 32.41 (13.8) ng/ml; placebo group: 36.18 (17.1) ng/ml	N/A	D3, 400 IU/day, 1,000 IU/day	Placebo	Oral	12 months	N/A
**Mammography density**											
Brisson et al. (25)	405(405)	42.7	46°48′N	Pre	Vit D group: 65.1 (24.7), 65.6 (25.4), 59.3 (21.0) nmol/L; placebo group: 65.7 (23.5) nmol/L	Vit D groups: 38.3 (14.5), 37.2 (15.2), 37.9 (15.8); placebo group: 40.8 (17.2)	D3, 1,000 IU/day, 2,000 IU/day, 3,000 IU/day	Placebo	Oral	12 months	Breast Imaging Reporting and Data System (BIRADS), semi-automated and automated methods
Crew et al. (39)	204(204)	44.6	N/A	Pre	Vit D group: 23.9 (7.2) ng/ml, placebo group: 23.7 (8.4) ng/ml	Vit D group: 38.6 (18.0); placebo group: 35.0 (19.0)	D3, 20,000 IU/week	Placebo	Oral	24 months	Semi-automated methods with the Cumulus software
Wood et al. (38)	300(300)	42.6 (6.4)	N/A	Pre	26.6 (11.7) ng/ml	49% of women had MD between 25 and 50% with only 12% over 50% dense	D3, 2,000 IU/day	Placebo	Oral	12 months	Digital computed radiography (CR) and digital direct radiography (DR)

*SD, standard deviation; MD, mammography density; RR, risk ratio*.

Regarding risk of bias assessment, three studies were graded as high risk of bias (38, 57, 58), because of incomplete outcomes due to unknown reasons or loss of follow-up (Supplementary Figures 1A,B).

### Effect of Vitamin D Supplementation on Risk of Breast Cancer

**Figure 2:**
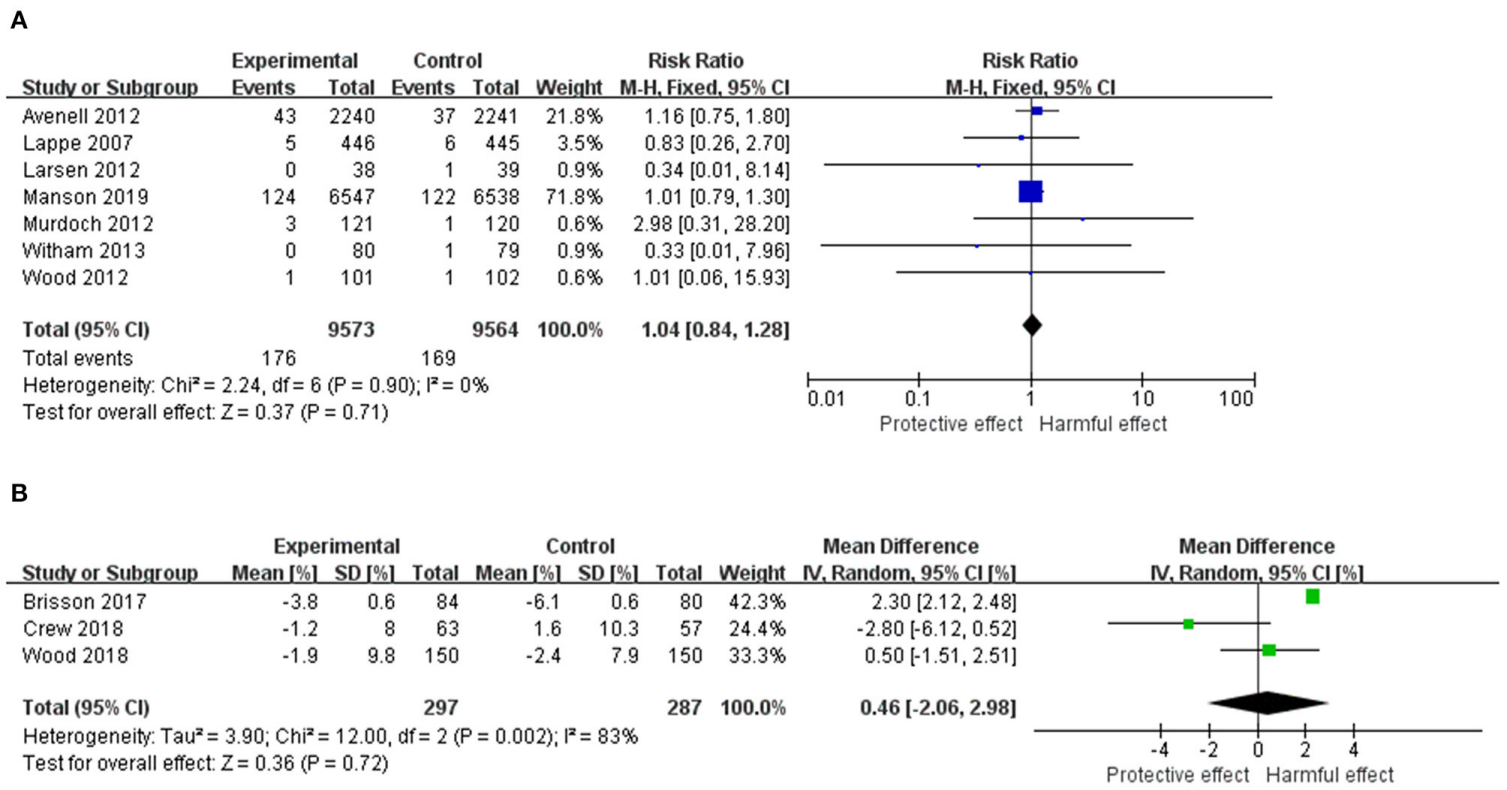
**(A)** Forest plot of the RR of breast cancer for Vitamin D supplementation vs. placebo. **(B)** Forest plot of the mean difference of MD change for Vitamin D supplementation vs. placebo. The size of the data markers (squares) for the RR/mean difference corresponds to the weight of the study in the meta-analysis; the horizontal lines correspond to the 95% CI values. RR, risk ratio; MD, mammography density.

The pooled results from the individual RCTs including 19,137 females are displayed in Figure 2A. There was no statistically significant effect of vitamin D supplementation (RR = 1.04, 95% CI: 0.84–1.28, *p* = 0.71). The heterogeneity among studies was not significant (*I*^2^ = 0%, χ^2^ = 2.24, *p* = 0.90).

**Figure 3:**
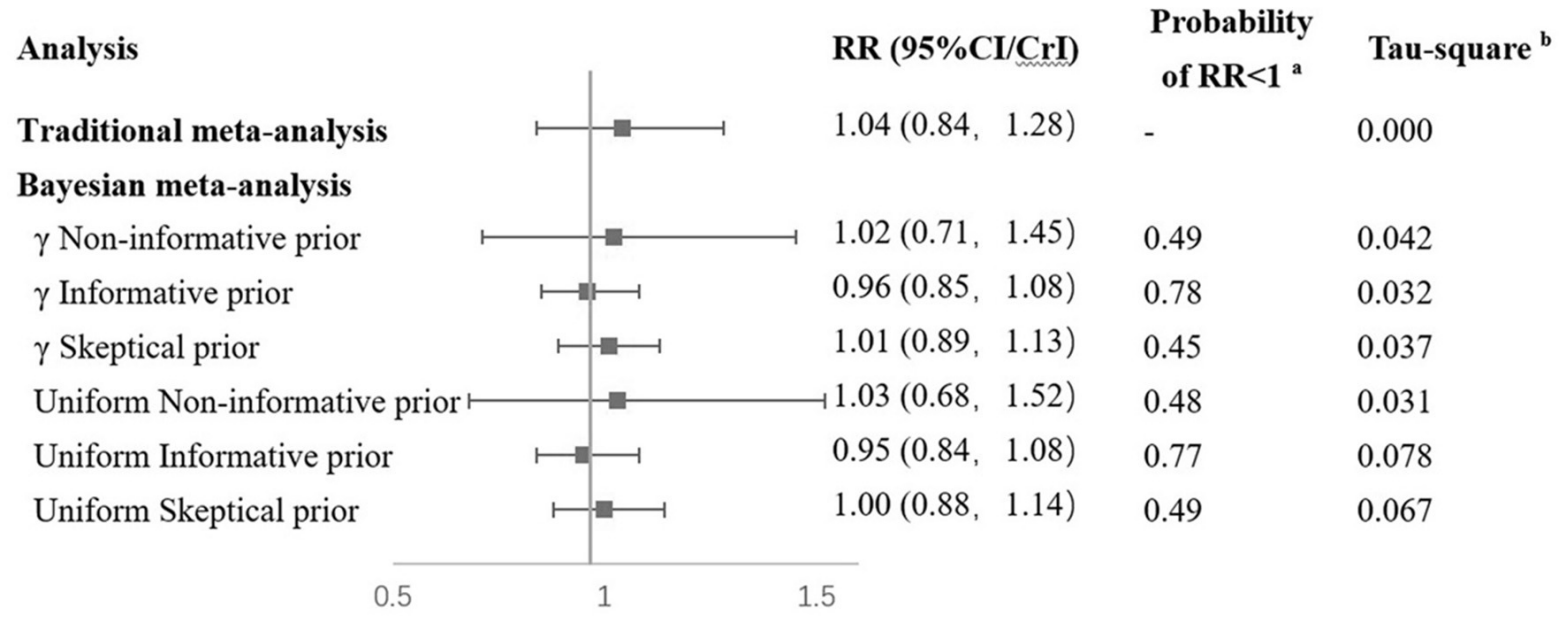
Results of combination of RCTs and observational studies in Bayesian. ^a^RR < 1 means that results favor Vitamin D supplementation. ^b^Tau-square means between study variance. RCT, randomized controlled trial; RR, risk ratio; MD, mammography density.

When the Bayesian approach was applied using γ distribution as non-informative prior distribution, the findings were similar to classical analysis results (Figure 3). With the use of informative prior distribution, the pooled RR was 0.96 (95% CrI: 0.85–1.08). The posterior probability of a protective effect of vitamin D supplementation was 0.78. With the use of skeptical prior distribution, the RR was 1.01 (95% CrI: 0.89–1.13), and the posterior probability of favoring vitamin D supplementation was 0.45, which is similar to the non-informative results. Sensitivity analyses using a different prior distribution (uniform distribution for the between-study SD) yielded similar results to the γ prior distribution (Figure 3).

None of the subgroup analyses showed a significant effect of vitamin D supplementation on risk of breast cancer (Table 2). For each subgroup, the heterogeneity was not significant (all *I*^2^ = 0%). The three sensitivity analyses also produced similar findings to the main results (Table 2).

**Table 2:** Subgroup analysis and sensitivity analysis for incidence of breast cancer.

**Analysis**	**No. of studies included**	**No. of patients/participants included**	**RR (95% CI)**	***p*-value**
**SUBGROUP ANALYSIS**				
**Different vitamin D dosage**
High^a^	3	251/13,403	1.02 (0.80, 1.31)	0.87
Low	4	94/5,734	1.09 (0.73, 1.63)	0.66
**Different follow-up duration**
Long^b^	3	261/14,217	1.02 (0.80, 1.30)	0.88
Short	4	84/4,920	1.11 (0.73, 1.69)	0.63
**Study location**
High latitude^c^	4	84/4,920	1.11 (0.73, 1.69)	0.63
Low latitude	2	15/1,132	1.09 (0.39, 3.11)	0.87
**Menopausal status**
Post-	5	95/5,811	1.07 (0.72, 1.60)	0.73
Pre-^d^	-	-	-	-
Unknown	2	250/13,326	1.03 (0.80, 1.32)	0.83
**Baseline vitamin D level**
High^e^	4	263/14,420	1.02 (0.80, 1.30)	0.88
Low	3	82/4,717	1.11 (0.72, 1.70)	0.63
**Sensitivity analysis**				
Excluding studies with high risk of bias	5	88/5,161	1.15 (0.76, 1.74)	0.52
Excluding studies that involved non-study cointerventions	4	8/680	1.00 (0.25, 3.97)	1.00
Using data of the lowest dose for each study	7	345/19,137	1.04 (0.84, 1.28)	0.71
Fixed-effects model	7	345/19,137	1.04 (0.84, 1.28)	0.71

*RR, risk ratio; CI, confidence interval*.

a *>1,500 IU/day*.

b *>12 months*.

c *>50°*.

d *No study that included premenopausal women was found*.

e *Serum 25(OH)D > 25 ng/ml*.

### Effect of Vitamin D Supplementation on Mammography Density

Results for the effect of vitamin D on MD including 584 females are shown in Figure 2B. There was no statistically significant effect of vitamin D supplementation on MD from baseline (mean difference = 0.46, 95% CI: −2.06–2.98, *p* = 0.72). The heterogeneity among studies was substantial (*I*^2^ = 83%, χ^2^ = 12.00, *p* = 0.002).

### Quality of a Body of Evidence

The quality of a body of evidence obtained from all the included trials for risk of breast cancer was graded as moderate, because of risk of incomplete outcome data in the included trials (Supplementary Table 2). The quality of a body of evidence for MD was graded as low, due to unexplained heterogeneity and risk of incomplete outcome data among the included studies (Supplementary Table 2).

### Assessment of Publication Bias

For both risk of breast cancer and MD, the symmetric funnel plot suggested no evidence of publication bias (see Supplementary Figure 1 for the funnel plot to assess publication bias). Egger's test and Begg's test yielded similar results to the visual inspection for symmetry of funnel plot, with no evidence of publication bias observed (*p*-values > 0.05).

## Discussion

### Main Finding

Seven RCTs were identified to investigate the effect of vitamin D supplementation on risk of breast cancer. No statistically significant effect was found in classical random-effects meta-analysis. The results were consistent in subgroup analyses and sensitivity analyses. When Bayesian meta-analyses were conducted, results remained non-significant with the use of non-informative, informative, or skeptical prior distributions. There was no statistically significant effect found on MD.

Laboratory works found that vitamin D could have a protective effect on breast cancer (13, 59–63); however, results from epidemiology studies remained conflicting. Observational studies found that using supplemental vitamin D was associated with reduced risk of overall or estrogen receptor (ER)-positive breast cancer, although some without statistical significance (18, 64–66). In RCTs, point estimates showed both positive and negative associations, but none of them was significant (52–58) (Figure 2). The non-significant results could be due to low endpoint serum 25(OH) levels in these studies. In a pooled analysis of two RCTs and a prospective cohort where a dose–response analysis was carried out, women with serum 25(OH)D concentrations over 60 ng/ml had significantly lower risk of breast cancer than those with concentrations <20 ng/ml (66); however in most of the previous studies, participants' average post-intervention 25(OH)D levels were lower than 50 ng/ml. A higher dosage of vitamin D supplementation than the existing RCTs, which could theoretically lead to a higher serum 25(OH)D level, may have a stronger effect on breast cancer. Nevertheless, the potential harmful effect of vitamin D overdose should not be neglected, especially in those who have sufficient vitamin D intake (67). Therefore, further evidence from high-quality and well-designed studies is needed to determine the optimal dosage of vitamin D supplementation for the prevention of breast cancer.

A Bayesian meta-analysis was conducted to combine the findings from both observational studies and RCTs. In the Bayesian analysis, a result using non-informative distribution was similar to that of traditional meta-analysis (RR = 1.02). When data from observational studies were introduced in informative prior distribution, the point estimation suggested a protective effect of vitamin D supplementation on breast cancer (RR = 0.96) with a posterior probability of a protective effect of vitamin D supplementation of 0.78, although without statistical significance (Figure 3). These results from the Bayesian analysis suggested that in the observational studies, the protective effect of vitamin D could be overestimated. In observational studies, the use of vitamin D supplementation was generally estimated from self-reports. The dosage, duration, and frequency could probably be less accurate than RCTs, especially in retrospective studies. Potential confounders such as latitude of study location were not properly adjusted in the existing observational studies, which could also introduce bias to their results.

Experimental studies suggested that the protective effect of vitamin D on breast cancer could be stronger in postmenopausal women (13, 59–63, 68), because their local estrogen synthesized in the breast microenvironment was the driver of the development of ER-positive breast cancer (68). However, in our analysis, the result in postmenopausal subgroup remained non-significant. This may suggest that the effect of oral vitamin D supplementation on breast cancer risk was not mediated by menopausal status.

### Comparison With Previous Reviews

In a previous review by Bjelakovic et al. (23) that synthesized evidence from RCTs (23), the pooled results of seven RCTs showed a non-significant protective effect of vitamin D (RR = 0.97, 95% CI: 0.86–1.09). Even though we included the most up-to-date evidence from recent trials for pooled analyses, our findings kept consistent with theirs regarding the lack of a statistically significant effect of vitamin D on breast cancer risk (Figure 2A). Therefore, more high-quality studies are needed to further clarify the effect of vitamin D supplementation on risk of breast cancer and to inform the decision to supplement vitamin D.

### Strengths and Limitations

Our current study is the most up-to-date review investigating the effect of vitamin D supplementation on breast cancer risk synthesizing RCTs, with a recent published large trial included. We performed a comprehensive and exhaustive search to retrieve all relevant studies and extracted and managed data in duplicate with a good level of consensus. Our study included a large and representative sample, therefore enhancing the generalizability of our findings. *A priori* and *post-hoc* subgroup analyses and sensitivity analyses were carried out to better combine the available evidence. The particular strength of the current review was the use of the Bayesian approach, which allowed us to utilize external information from previous observational studies for our meta-analysis of RCTs and to explore the robustness of our results under different assumptions (i.e., with different prior distributions).

There are several limitations to this systematic review and meta-analysis. First, vitamin D is proved to inhibit both ER-positive and ER-negative breast cancer cells, while the effect on ER-positive cancer cells is stronger (69). Unfortunately, none of the included RCTs reported the type of their breast cancer cases; therefore, a subgroup analysis stratified by the cancer types was not able to be conducted. Besides, for the primary outcome, four out of seven studies had study durations <12 months, which is a relatively short period for progression of cancer. Third, for the geographical distribution of the included studies, all of them were carried out in developed countries, while the mortality rate of breast cancer is still increasing mainly in developing countries. For example, in 2008, almost 50% of breast cancer cases and 58% of deaths occurred in developing countries^1^. The aforementioned weaknesses to the design of current studies would limit the generalizability and weaken the clinical significance. Moreover, Avenell's and Manson's studies had higher weights than other studies, which therefore dominated the findings from a pooled analysis. However, after carefully comparing these two studies with the remaining trials, a similarity between them was found with a non-significant heterogeneity detected (*I*^2^ = 0%). Furthermore, we used the trim-and-fill technique as a sensitivity analysis by excluding either Avenell's (RR = 1.01, 95% CI: 0.79–1.28, *p* = 0.96) or Manson's (RR = 1.11, 95% CI: 0.75–1.64, *p* = 0.61) studies at one time. Results consistent to the global analysis (Figure 2A) were found. Meanwhile, the heterogeneity among studies for the secondary outcome was statistically significant (*p* = 0.002, *I*^2^ = 83%). However, the number of included RCTs was small (*n* = 3), which precluded our further exploration on their heterogeneity. Three out of 10 included studies were graded as high risk of bias, mainly because of inconsistency due to no clear reason of loss of follow-up. Small sample size, substantial heterogeneity or the secondary outcome, and high risk of bias in some of the studies downgraded the quality of the analysis.

### Clinical Implication of the Study

The current evidence does not support the effect of vitamin D supplementation on decreased breast cancer risk. Although the observational studies found a significant protective effect of vitamin D on breast cancer, the Bayesian analysis implicated that such results from observational studies could be overestimated. However, some evidence has suggested the protective role of vitamin D in the progression and prognosis of breast cancer. For example, vitamin D was associated with increased effectiveness of anticancer drugs, reduced risks of metastasis and recurrence, and superior survival in patients (70, 71). Besides, epigenetics modulations induced by vitamin D for breast cancer patients had been reported (72, 73). Therefore, further interventional studies may be needed to clarify efficacy of vitamin D supplementation in both the prevention and treatment of breast cancer, using higher dosage of vitamin D, in participants of different MD and menopausal status, and especially in developing countries.

### Conclusions

In conclusion, our systematic review and meta-analysis found that there was a lack of evidence to support the efficacy of vitamin D supplementation in breast cancer risk and change of MD. The protective effect of vitamin D on risk of breast cancer from previous observational studies may be overestimated. Further high-quality RCTs with large sample size and longer study duration are needed to clarify the efficacy the efficacy of vitamin D in breast cancer.

## Data Availability Statement

The original contributions presented in the study are included in the article/Supplementary Material, further inquiries can be directed to the corresponding author.

## Author Contributions

Conceptualization: ZL, LW, GL, and LT. Data collection and analyses: ZL, LW, and GL. Writing—original draft preparation: ZL. Writing—review and editing. ZL, XH, JZ, GL, and LT. GL had primary responsibility for final content. All authors contributed to the article and approved the submitted version.

## Conflict of Interest

The authors declare that the research was conducted in the absence of any commercial or financial relationships that could be construed as a potential conflict of interest.

## Footnotes

^1^World Health Organization. Breast cancer: prevention and control. Available from: https://www.who.int/cancer/detection/breastcancer/en/index1.html (accessed November 30, 2019).

## Abbreviations

25(OH)D: 25-hydroxyvitamin D
RCT: randomized controlled trial
RR: risk ratio
CI: confidence interval
MD: mammography density
PRISMA: preferred reporting items for systematic reviews and meta-analyses
CENTRAL: cochrane central register of controlled trials
WMD: weighted mean difference
CrI: credible interval
GRADE, grading of recommendations assessment: development and evaluation
ER: estrogen receptor
SD: standard deviation.
