# Supplementary material for: Effect of Vitamin D Supplementation on Risk of Breast Cancer: A Systematic Review and Meta-Analysis of Randomized Controlled Trials
Source: Front Nutr. 2021 Apr 1;8:655727. doi: 10.3389/fnut.2021.655727 (PMC8049142; doi:10.3389/fnut.2021.655727)
Supplement: Supplementary file 1 [file Data_Sheet_1.docx]

**Table S1** Search terms modified for Medline and EMBASE (from Jan 1st, 1946 to Jun 10th, 2019)

| **Steps** | **Search terms** |
| --- | --- |
| 1 | exp Vitamin D/ |
| 2 | (Vitamin D or vitamin D2 or vitamin D3 or 1-alpha hydroxyvitamin D3 or 1-alpha-hydroxy-vitamin D3 or 1-alpha hydroxycalciferol or 1-alpha-hydroxy-calciferol or 1,25 dihydroxyvitamin D3 or 1,25-dihydroxy-vitamin D3 or 1,25 dihydroxycholecalciferol or 1,25-dihydroxycholecalciferol or 25-hydroxycholecalciferol or 25 hydroxycholecalciferol |
| 3 | 1 or 2 |
| 4 | exp Breast Neoplasms/ |
| 5 | (Breast cancer or breast tumour or breast tumor or breast oncology or breast carcinoma or breast neoplasm or mammary cancer or mammary tumour or mammary tumor or mammary oncology or mammary carcinoma or mammary neoplasm).mp. [mp=title, abstract, heading word, drug trade name, original title, device manufacturer, drug manufacturer, devi |
| 6 | exp Breast Density/ or exp Mammography/ |
| 7 | (breast density or mammary density or mammography or mammogram or mammographic density or mammographic image).mp. [mp=title, abstract, heading word, drug trade name, original title, device manufacturer, drug manufacturer, device trade name, keyword, floating subheading word, candidate term word] |
| 8 | 4 or 5 or 6 or 7 |
| 9 | exp Clinical Trial Protocol/ or exp Pragmatic Clinical Trial/ or exp Clinical Trial, Phase II/ or exp Clinical Trial Protocols as Topic/ or exp Controlled Clinical Trial/ or exp Clinical Trial, Phase I/ or exp Randomized Controlled Trial/ or exp Clinical Trial, Veterinary/ or exp Clinical Trial/ or exp Clinical Trial, Phase III/ or exp |
| 10 | (trial or clinical trial or random* controlled trial or RCT or group* or experiment* or intervention or placebo).mp. [mp=title, abstract, heading word, drug trade name, original title, device manufacturer, drug manufacturer, device trade name, keyword, floating subheading word, candidate term word] |
| 11 | 9 or 10 |
| 12 | 3 and 8 and 11 |
| 13 | limit 12 to human |

**Figure S1a** Risk of bias graph: review authors' judgements about each risk of bias item presented as percentages across all included studies that breast cancer risk is outcome


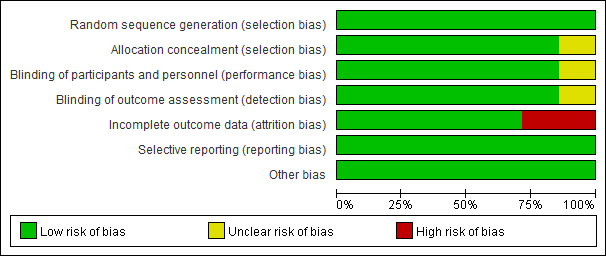


**Figure S1b** Risk of bias graph: review authors' judgements about each risk of bias item presented as percentages across all included studies that breast cancer risk is outcome


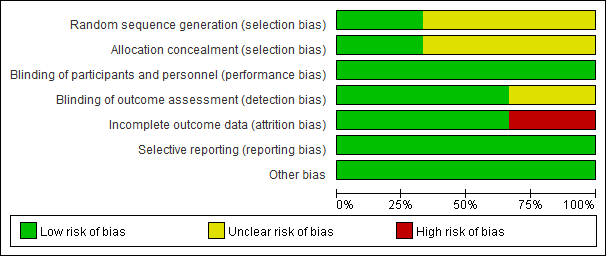


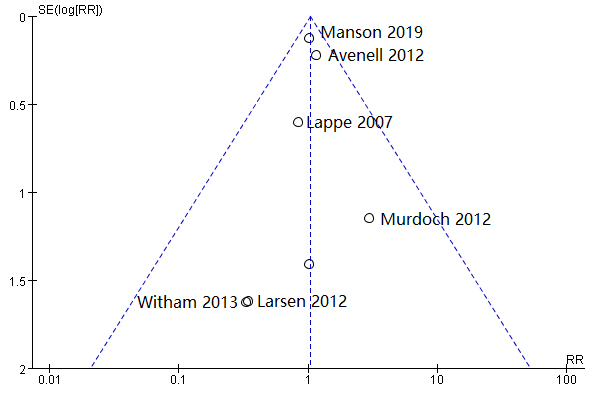
**Figure S2a** Funnel plot to assess publication bias for efficacy of Vitamin D supplementation on risk of breast cancer


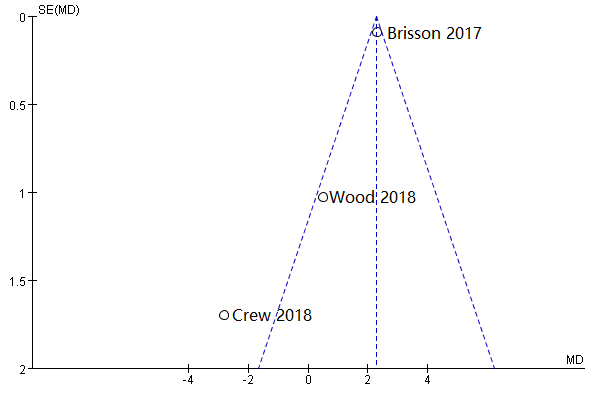
**Figure S2b** Funnel plot to assess publication bias for efficacy of Vitamin D supplementation on mammographic density

**Table S2** Summary of findings for efficacy of Vit D supplementation on risk of breast cancer

| **Patient or population: Female without breast cancer at baseline Settings: Multicenter, multinational data from RCT** | | | | | | |
| --- | --- | --- | --- | --- | --- | --- |
| **Intervention: Vitamin D supplementation Comparison: placebo** | | | | | | |
| **Outcomes** | **Illustrative comparative effect* (95% CI)** | | **Relative effect** | **No of Participants** | **Quality of the evidence** | **Comments** |
|  | **Assumed risk** | **Corresponding risk** | **(95% CI)** | **(studies)** | **(GRADE)** |  |
|  | Vitamin D | Placebo |  |  |  |  |
| *Results of vitamin D s uplmentation and breast cancer risk* | | | | | | |
| **Incidence of breast cancer** | Incident rate ranging from 0 to 2.48% per 100 patient-years | Incident rate ranging from 0.83% to 2.56% per 100 patient-years | 1.04 | 19,137 | ⊕⊕⊕⊝^1^ | - |
| Follow-up: varied from 4 months to 5 years |  |  | (0.48 – 1.28) | (7 studies) | Moderate |  |
| **Mammographic density** | Change in MD ranging from -3.8% to -1.2% | Change in MD ranging from -6.1% to 1.6% | 0.46 | 584 | ⊕⊕⊝⊝^2^ | - |
| Follow-up: varied from 12 to 24 months |  |  | (-2.06 to 2.98) | (3 studies) | Low |  |
|  |  |  |  |  |  |  |
| *The basis for the assumed risk (e.g. the median control group risk across studies) is provided. The corresponding risk (and its 95% confidence interval) is based on the assumed risk in the comparison group and the relative effect of the intervention (and its 95% CI). | | | | | | |
| CI: Confidence interval; MD: Mammographic Density; RCT: randomized controlled trial | | | | | | |
| GRADE Working Group grades of evidence | | | | | | |
| High quality: Further research is very unlikely to change our confidence in the estimate of effect. | | | | | | |
| Moderate quality: Further research is likely to have an important impact on our confidence in the estimate of effect and may change the estimate. | | | | | | |
| Low quality: Further research is very likely to have an important impact on our confidence in the estimate of effect and is likely to change the estimate. | | | | | | |
| Very low quality: We are very uncertain about the estimate. | | | | | | |

^1^ Due to the risk of bias in the included studies

^2^ Due to the risk of bias in the included studies and inconsistency
